# Microbial transformation of the Deepwater Horizon oil spill—past, present, and future perspectives

**DOI:** 10.3389/fmicb.2014.00603

**Published:** 2014-11-18

**Authors:** Nikole E. Kimes, Amy V. Callaghan, Joseph M. Suflita, Pamela J. Morris

**Affiliations:** ^1^Evolutionary Genomics Group, División de Microbiología, Universidad Miguel HernándezSan Juan, Spain; ^2^Department of Microbiology and Plant Biology, University of OklahomaNorman, OK, USA; ^3^Belle W. Baruch Institute for Marine and Coastal Sciences, University of South CarolinaGeorgetown, SC, USA

**Keywords:** Deepwater Horizon, Gulf of Mexico, microbial response to oil spill, microbial degradation of hydrocarbons, aerobic hydrocarbon degradation, anaerobic hydrocarbon degradation, oxyhydrocarbons

## Abstract

The Deepwater Horizon blowout, which occurred on April 20, 2010, resulted in an unprecedented oil spill. Despite a complex effort to cap the well, oil and gas spewed from the site until July 15, 2010. Although a large proportion of the hydrocarbons was depleted via natural processes and human intervention, a substantial portion of the oil remained unaccounted for and impacted multiple ecosystems throughout the Gulf of Mexico. The depth, duration and magnitude of this spill were unique, raising many questions and concerns regarding the fate of the hydrocarbons released. One major question was whether or not microbial communities would be capable of metabolizing the hydrocarbons, and if so, by what mechanisms and to what extent? In this review, we summarize the microbial response to the oil spill as described by studies performed during the past four years, providing an overview of the different responses associated with the water column, surface waters, deep-sea sediments, and coastal sands/sediments. Collectively, these studies provide evidence that the microbial response to the Deepwater Horizon oil spill was rapid and robust, displaying common attenuation mechanisms optimized for low molecular weight aliphatic and aromatic hydrocarbons. In contrast, the lack of evidence for the attenuation of more recalcitrant hydrocarbon components suggests that future work should focus on both the environmental impact and metabolic fate of recalcitrant compounds, such as oxygenated oil components.

## Introduction—the deepwater horizon (DWH) blowout and the resulting oil spill

The Macondo 252 well, located ~45 miles off the Louisiana coast in the Gulf of Mexico, suffered a catastrophic blowout on April 20, 2010, which resulted in the tragic death of 11 workers. After continuing to burn for 2 days, the mobile offshore drilling unit, the Deepwater Horizon (DWH), sank on April 22, 2010. Over the next 3 months, an estimated 4.1–4.4 million barrels of crude oil (Crone and Tolstoy, 2010; OSAT-I, 2010) and a disputed amount of gaseous hydrocarbons (Joye et al., 2011a,b; Kessler et al., 2011a,b) flowed into the depths (~1500 m) of the Gulf of Mexico. This unprecedented discharge of oil and gas, representing the largest oil spill in US history, resulted in a challenging and complex response effort to cap the wellhead on July 15, 2010, eighty-three days after the oil spill began. By August of 2010, the US federal government's National Incident Command estimated that 78% of the oil had been depleted through either human intervention (direct recovery from the well, 17%; chemically dispersed, 16%; burning, 5%; skimmed, 3%) or natural means (evaporated/dissolved, 24%; naturally dispersed, 13%). These estimates indicated that 22% of the spilled oil, in addition to the gas released, remained unaccounted for and either remained in the water column and mixed with sand and sediment or was metabolized by microorganisms (Ramseur, 2010). Over the past four years, numerous studies have focused on determining the fate and ecological impact of the oil and gas that flowed into the Gulf of Mexico following the DWH oil spill. Here, we present a review of some of the key findings (Table S1), specifically addressing the microbial response to this event.

## Microbial degradation of hydrocarbons (Figure 1)—past perspective

**Figure 1:**
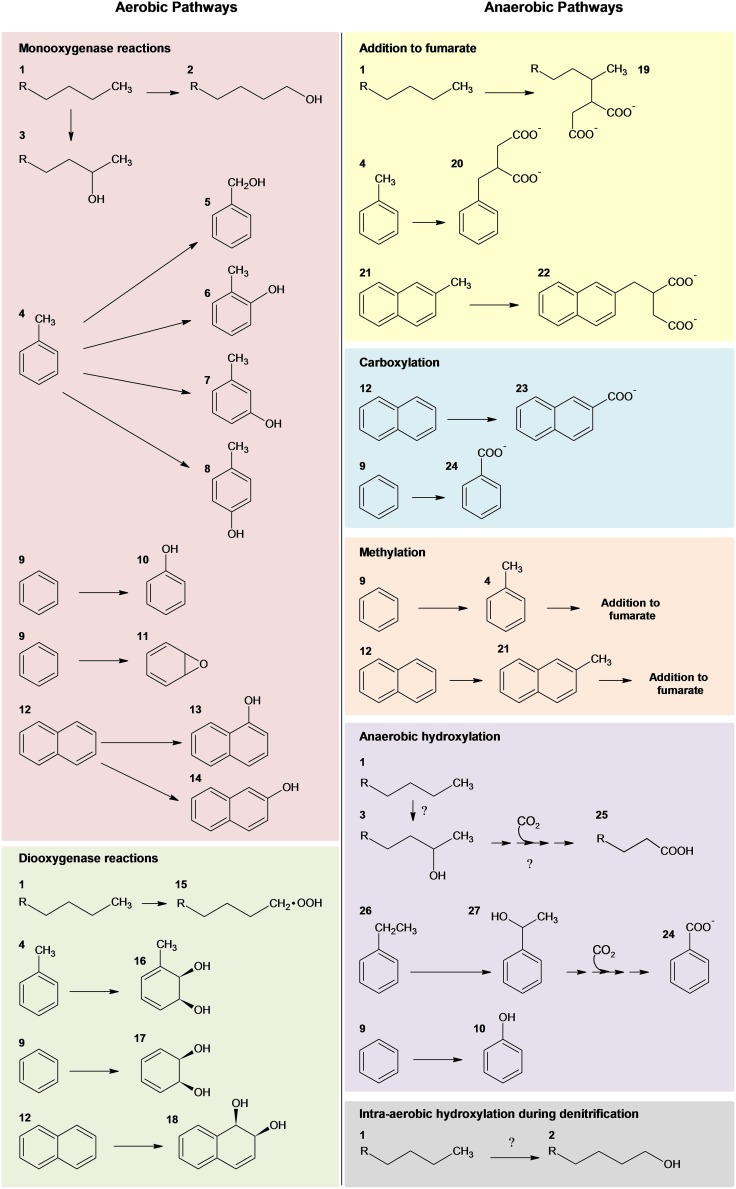
**Summary of microbial strategies for aerobic and anaerobic activation of hydrocarbons adapted from several reviews (Gibson and Parales, 2000; Parales et al., 2008; Pérez-Pantoja et al., 2010; Rojo, 2010; Austin and Callaghan, 2014; Callaghan, 2013b)**. Nomenclature: 1, *n*-alkane; 2, 1-alkanol; 3, 2-alkanol; 4, toluene; 5, benzyl alcohol; 6, *o*-cresol; 7, *m*-cresol; 8, *p*-cresol; 9, benzene; 10, phenol; 11, benzene epoxide; 12, naphthalene; 13, 1-naphthol; 14, 2-naphthol; 15, alkyl hydroperoxide; 16, toluene *cis*-1,2-dihydrodiol; 17, *cis*-1,2-dihydroxy-cyclohexadiene; 18, *cis*-1,2-dihydroxy-1,2-dihydronaphthalene; 19 –2-methylalkylsuccinate; 20, benzylsuccinate; 21, 2-methylnaphthalene; 22, naphthyl-2-methylsuccinate; 23, 2-naphthoate; 24, benzoate; 25, *n*-fatty acid; 26, ethylbenzene; and 27, 1-phenylethanol; Question marks designate recent hypothesized pathways of anaerobic alkane degradation (Zedelius et al., 2011; Sünwoldt et al., 2012; Heider and Schühle, 2013).

Over the last century, substantial advances in our knowledge of the microbial degradation of hydrocarbons has occurred (Atlas et al., 2011). Since the first isolation of hydrocarbon-degrading bacteria in 1913 (Söhngen, 1913), over 79 genera of bacteria capable of utilizing hydrocarbons as a sole source of energy have been identified in addition to others that can degrade or transform hydrocarbons (Head et al., 2006; Prince et al., 2010). Marine environments alone harbor at least 25 genera of hydrocarbon-degrading bacteria, and these microbial communities are thought to be the primary organisms responsible for the attenuation of pollutants (Das and Chandran, 2011). The diversity of hydrocarbon compounds associated with oil (i.e., *iso*-, cyclo, and linear alkanes, monoaromatic compounds, and polycyclic aromatic hydrocarbons) necessitates different microorganisms with specific biochemical mechanisms directed at the metabolism of the various classes of hydrocarbon compounds (Timmis et al., 2010). Typically, an individual microorganism will biodegrade a limited number of hydrocarbons, whereas, microbial communities can biodegrade an impressive array of hydrocarbons collectively. Although **aerobic biodegradation of hydrocarbons** represents the most rapid and well known of these processes (Fritsche and Hofrichter, 2008), anaerobic degradation has also been well-characterized (Widdel et al., 2010; Heider and Schühle, 2013) and is especially important in oil-contaminated marine environments (Head et al., 2006). A general overview of aerobic and anaerobic hydrocarbon degradation pathways is depicted in Figure 1.

KEY CONCEPT 1 **Aerobic microbial degradation of hydrocarbons**

Numerous microorganisms, predominantly of the bacterial phylum Proteobacteria, are capable of rapidly degrading a subset of hydrocarbon compounds via aerobic pathways. The initial oxidative step is achieved via mono- and dioxygenases. Subsequent peripheral pathways further degrade the compounds into intermediates of the central metabolic pathways (Figure 1).

The majority of aerobic oil-degrading bacteria described to date are alpha-, beta-, and gamma-proteobacteria (Head et al., 2006; van Beilen and Funhoff, 2007; Kim and Kwon, 2010). However, isolates of Actinomycetales, *Bacillus*, *Geobacillus*, and *Thermas* have also been shown to utilize alkanes (see van Beilen and Funhoff, 2007). Aerobes initiate biodegradation via mono- and dioxygenase enzymes (Haddock, 2010; Pérez-Pantoja et al., 2010; Rojo, 2010; Austin and Groves, 2011; Austin and Callaghan, 2014; Figure 1). One of the most well studied oil-degrading microorganisms is *Alcanivorax borkumensis*, which is a marine gammaproteobacterium known to utilize a broad range of aliphatic hydrocarbons (Schneiker et al., 2006; Yakimov et al., 2006; dos Santos et al., 2010) through multiple routes of terminal oxidation via several hydroxylating enzymes (i.e., *alkB1*, P450 cytochrome monooxygenase, and a putative flavin-binding monooxygenase) (Sabirova et al., 2006). Also important in marine ecosystems, especially marine sediments, is the **anaerobic biodegradation of hydrocarbons** (Coates et al., 1997; Davidova et al., 2007). Anaerobic microorganisms, including sulfate reducers, denitrifying bacteria, nitrate ammonifying bacteria, phototrophs, metal ion reducers, and methanogenic consortia, are capable of metabolizing a variety of hydrocarbons, ranging from *n*-alkanes and *n*-alkenes to the more recalcitrant aromatic compounds (Boll and Heider, 2010; Widdel and Grundmann, 2010; Widdel et al., 2010; Heider and Schühle, 2013) (Figure 1). To date, the most well characterized anaerobic mechanism for hydrocarbon activation and degradation is hydrocarbon addition to fumarate (for review and references within see Heider and Schühle, 2013). Alternative anaerobic mechanisms, however, have also been described, including intra-aerobic hydroxylation, oxygen-independent hydroxylation, and carboxylation (for reviews see Callaghan, 2013a; Heider and Schühle, 2013).

KEY CONCEPT 2 **Anaerobic microbial degradation of hydrocarbons**

A diverse array of anaerobic microorganisms (e.g., sulfate reducers, denitrifying bacteria, nitrate ammonifying bacteria, phototrophs, metal ion reducers, and methanogenic consortia) can metabolize hydrocarbons, including the more recalcitrant aromatic compounds. Hydrocarbon activation is achieved through a variety of mechanisms, including hydrocarbon addition to fumarate, intra-aerobic hydroxylation, oxygen-independent hydroxylation, and carboxylation (Figure 1).

The ability of microbial communities to metabolize hydrocarbons is not surprising given the ubiquitous distribution of hydrocarbons in the environment resulting from both natural and anthropogenic inputs. The National Research Council (2003) reported that up to 47% of the crude oil entering marine habitats is the result of natural oil and gas seeps, which provide a consistent and long-term adaptive pressure to microbial communities in these regions. Satellite imagery of the Gulf of Mexico provides the only overall estimates to date and suggests a yearly flow rate of 120,000 barrels/day (MacDonald et al., 1993), if not more in some regions (Hu et al., 2009). This level of exposure results in microbial communities capable of hydrocarbon degradation (Kappell et al., 2014). For example, the Gulf of Mexico seafloor sediments near cold seeps contain high levels of sulfate-reducing bacteria (i.e., Deltaproteobacteria) and anaerobic methanotrophs (i.e., ANME archaea), which are presumed to be involved in the biodegradation of both methane and non-methane hydrocarbons (Orcutt et al., 2010). Despite the advancements made over the last century in characterizing hydrocarbon-metabolizing microbial communities, the DWH spill was an unprecedented incident, and as such, it provided a unique research opportunity to better understand the role of indigenous microbial communities in the bioremediation of hydrocarbons under the prevailing circumstances (Table S1). We summarize the response of microbial communities to the DWH oil spill as evidenced by numerous studies in the literature in an attempt to determine if the lessons of past hydrocarbon spills can be extrapolated to this one. As a result, we propose a shift in the focus, or “starting-point,” of subsequent studies in the case of future oil spills.

## Microbial hydrocarbon degradation following the DWH oil spill (Figure 2)

### The water column

The MC252 oil flowing from the DWH spill was a light crude oil that consisted predominantly of alkanes (i.e., saturated hydrocarbons), but also contained 16% aromatic hydrocarbons and 10% polar compounds (Reddy et al., 2012). Within a month of the DWH spill, a deep water oil plume consistent with dispersed MC252 oil was detected at ~1100 meters depth (Camilli et al., 2010; Hazen et al., 2010). The plume contained a complex mixture of hydrocarbons including alkanes, monoaromatic hydrocarbons (e.g., BTEX) and polycyclic aromatic hydrocarbons (i.e., PAHs) (Diercks et al., 2010; Reddy et al., 2012), and it is now routinely distinguished through the use of known biomarkers and their respective ratios. In addition, natural gas components such as methane, ethane and propane were also detected at significant, but debatable, levels (Valentine et al., 2010; Joye et al., 2011a,b; Kessler et al., 2011a,b; Reddy et al., 2012). This complex mixture of hydrocarbons released at depth and in cold waters (4–6°C) resulted in an increase in microbial biomass within the plume (Hazen et al., 2010). The indigenous microbial community also underwent a phylogenetic shift that was specific to the deep water plume, showing significant variation and loss of diversity compared to both the non-plume communities at depth and the surface oil slick communities (Redmond and Valentine, 2012; Yang et al., in press). Furthermore, this shift was dynamic, and the **succession of dominant bacteria** present at any given location was reflective of the corresponding availability of hydrocarbon compounds (i.e., initially high levels of saturated hydrocarbons declined after the well was capped and sealed, which led to the relative increase in the more recalcitrant aromatic hydrocarbons, Dubinsky et al., 2013). The use of ^13^C-labeled hydrocarbons and isolation methodologies provided additional evidence indicating that the shifting communities from the affected water column were capable of degrading multiple classes of hydrocarbons (Gutierrez et al., 2013).

KEY CONCEPT 3 **Successional changes in indigenous microbial community composition**

**Figure 2:**
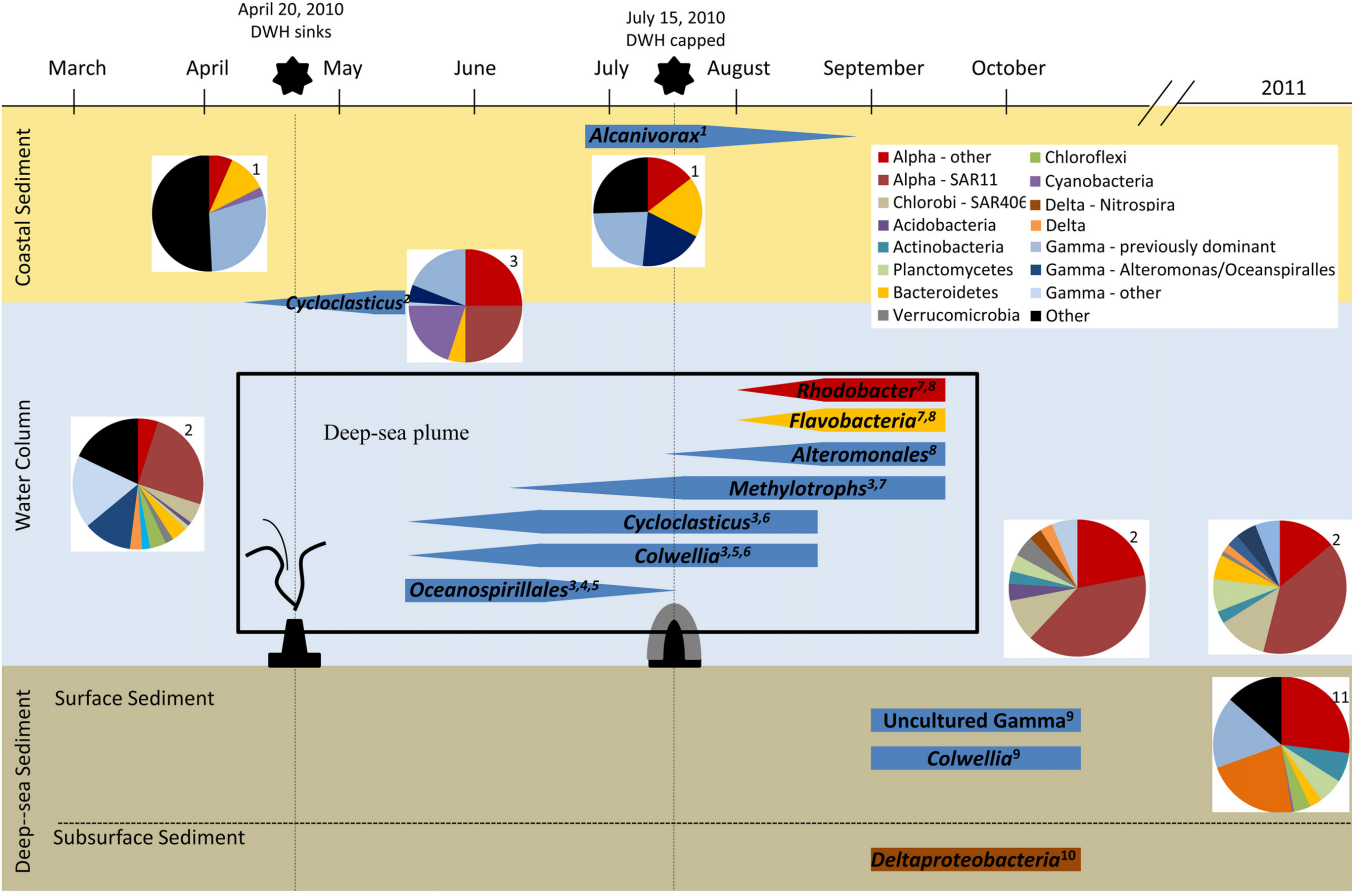
**Overview of microbial response to the DWH oil spill**. Data from numerous studies following the DWH oil spill are represented in this schematic using approximations from the published data referenced. 1, Kostka et al., 2011; 2, Yang et al., in press; 3, Redmond and Valentine, 2012; 4, Hazen et al., 2010; 5, Mason et al., 2012; 6, Valentine et al., 2010; 7, Kessler et al., 2011b; 8, Dubinsky et al., 2013; 9, Mason et al., 2014b; 10, Kimes et al., 2013; 11, Liu and Liu, 2013.

The Gulf of Mexico harbors abundant and diverse microbial communities, including aerobic and anaerobic hydrocarbon degraders, associated with diverse physical niches (e.g., surface water, deep water column, deep sea sediments, and coastal sediments). Studies following the DWH oil spill established that these communities underwent shifts in composition that reflected the availability of specific hydrocarbon compounds (**Figure 2**).

Based on 16S rRNA studies, the plume-related microbial communities sampled from late May to early June 2010 (earliest samples following the DWH spill reported to date) showed an overwhelming dominance of a novel, uncultured gammaproteobacterium in the order Oceanospirillales (Hazen et al., 2010; Redmond and Valentine, 2012). Metagenomic and metatranscriptomic data provided additional evidence that Oceanospirillales was not only the dominant microorganism present in late May, but that it was also the most active group (Mason et al., 2012). Although GeoChip 4.0 evidence suggested the enrichment of genes involved in both aerobic and anaerobic hydrocarbon degradation in the plume (Lu et al., 2012), metatranscriptomic data from samples taken on the same cruise suggested that the aerobic degradation of hydrocarbons was the more dominant *in situ* process at that point in time (Mason et al., 2012). Genes involved in aerobic alkane degradation (e.g., alkane monooxygenase, cyclohexanol dehydrogenase, and cyclohexanone monooxygenase) were expressed at significantly higher levels in the plume metatranscriptomes and were also present in the single-cell genomes of Oceanospirillales, whereas transcripts involved in aromatic hydrocarbon degradation were either detected at lower levels or not at all (Mason et al., 2012).

By mid- to late June 2010, the microbial community composition in the water column had shifted and was dominated by two alternative gammaproteobacteria groups, *Cycloclasticus* and *Colwellia* (Valentine et al., 2010; Redmond and Valentine, 2012). Both genera are known psychrophiles, and some *Cycloclasticus* bacteria are capable of aerobically degrading aromatic hydrocarbons (Kasai et al., 2002). Microcosm experiments utilizing the oil-spill plume samples, however, revealed that *Colwellia* spp. were the dominant members in crude oil enrichments (i.e., methane, ethane, propane, and benzene), leading to speculation that *Colwellia* played a crucial role in the *in situ* degradation of ethane and propane during this time period (Redmond and Valentine, 2012). Similarly, oil (Macondo MC252) and dispersant (COREXIT 9500) enrichments of uncontaminated deep water samples resulted in the increased abundance of *Colwellia* (barely detectable to 15–30%), and to a much lesser degree (<1 to 5–10%) Oceanospirillales (Bælum et al., 2012). The single-cell genome of a *Colwellia* cell isolated from the DWH plume further suggests that these particular microorganisms may have supplanted the prior Oceanospirillales due to their capacity for gaseous and aromatic hydrocarbon degradation (Mason et al., 2014a). Interestingly, transcriptomic data indicated that *Colwellia* was indeed active, although not abundant, in the plume during late May 2010, prior to the observed *Colwellia* blooms in June 2010 (Mason et al., 2012).

Following the capping of the well, September 2010 water samples revealed another dramatic shift in the microbial community in which the previously identified groups (*Oceanospirillales*, *Cycloclasticus*, and *Colwellia*) had all but disappeared and were replaced by a more diverse community structure. The post-capping plume community included previously undetected methylotrophic bacteria (*Methylococcaceae, Methylophaga, and Methylophilaceae*), in addition to *Flavobacteria* and *Rhodobacterales* (Kessler et al., 2011b; Redmond and Valentine, 2012). The increased abundance of methylotrophic bacteria was particularly striking due to the low levels of methane and methane oxidation observed in the September plume (Kessler et al., 2011b), as well as the enrichments performed with September plume samples (Redmond and Valentine, 2012). Microbial methane degradation is a tightly coupled process initiated when methane is oxidized to methanol by methane oxidizers, such as *Methylococcaceae*, and further degraded by secondary consumers of C1 compounds, such as *Methylophaga* or *Methylophilaceae* (Dumont et al., 2011). As a result, it was postulated that the higher ratios of *Methylophaga* and *Methylophilaceae* to *Methylococcaceae* observed in the September samples (Kessler et al., 2011b; Redmond and Valentine, 2012) were the result of this process being observed at a later stage following an earlier bloom (Kessler et al., 2011b; Redmond and Valentine, 2012). This presumption was supported by high levels of methane oxidation measured within the plume from early June to mid-July (Dubinsky et al., 2013). However, Joye et al. (2011a) argued that the bloom in methylotrophs observed by Kessler et al. (2011b) was not statistically significant; furthermore, they suggested that the observed increase could have also resulted from methane added to the system during the response to the oil spill or from the degradation of alternative methylated compounds. Further evidence from August 2010 suggested that bacterial groups enriched in areas of oxygen anomalies, such as *Flavobacteria*, *Rhodobacteraceae*, and *Alteromonadaceae*, were responsible for scavenging the remnants of the bloom as relative levels of soluble hydrocarbons (e.g., BTEX) increased (Dubinsky et al., 2013). Community composition analysis (i.e., clone libraries and pyrosequencing) from post-plume samples collected near the wellhead in October 2010 and July 2011 revealed that the microbial community had returned to something similar to the pre-spill pelagic community (800 m, March 2010) (Yang et al., in press). It should be noted here that several of the early studies conducted after the spill did not employ next-generation sequencing technologies (see Table S1), and as such, some of the observed phylogenetic differences may have been related to lower levels of coverage obtained using alternative sequencing methods.

### Surface water

In addition to the deep water plume that formed following the DWH oil spill, numerous oil slicks were observed on the Gulf of Mexico surface (i.e., surface slicks) that contained high concentrations of *n*-alkanes (Hazen et al., 2010) and polycyclic aromatic hydrocarbons (Camilli et al., 2010; Diercks et al., 2010; Hazen et al., 2010). Interestingly, 16S rRNA gene clone libraries and pyrosequencing from a surface slick located near the wellhead in early May 2010 revealed a microbial community dominated by gammaproteobacteria, similar to the phenomenon noted in the deep water plume. However, in the case of the surface slick, *Cycloclasticus* was detected, a genus known to degrade aromatic hydrocarbons (Yang et al., in press). Isolation and enrichment experiments also identified *Cycloclasticus* as the dominant microbial group involved in aromatic hydrocarbon degradation from the same surface samples, providing compelling evidence of their functionality (Gutierrez et al., 2013). Pyrosequencing of oil mousses from the sea surface at three distinct locations on May 10, 2010 provided a different picture in which more diverse communities were dominated (>65%) by either alpha or gammaproteobacteria (Liu and Liu, 2013). Similarly, additional clone library studies from a surface slick sampled in late May 2010 revealed surface communities from 4 of 5 samples that were dominated by alpha and gammaproteobacteria and Cyanobacteria. One divergent sample revealed a community comprised exclusively of gammaproteobacteria, however, and neither *Cycloclasticus* nor Oceanospirillales represented a dominant group within this clone library (Redmond and Valentine, 2012). These data provided evidence that the surface slick communities were distinct from those found in the deep water plume, yet they likely experienced similar shifts in dominant microbial groups depending on the hydrocarbons that were present and bioavailable at a particular point in time and location.

### Deep-sea sediments

Although much of the oil and gas from both natural seeps and anthropogenic spills can persist in the water column or rise to the water surface as discussed above, it has also been shown that substantial amounts of water-soluble and/or particle-associated hydrocarbons also settle in deep-sea sediments (Jernelöv and Lindén, 1981; Ramseur, 2010). Deep-sea sediments, similar to the water column, harbor diverse and abundant microbial communities (Kallmeyer et al., 2012) that exhibit broad metabolic capabilities (D'Hondt et al., 2004). In the Gulf of Mexico, these communities, which are regularly exposed to hydrocarbons from natural cold-water seeps, are capable of degrading numerous hydrocarbon compounds (Joye et al., 2004; Lloyd et al., 2006; Orcutt et al., 2010). The predominant processes that contribute to this degradation can vary depending on numerous factors, such as the depth of the sediment examined, and include both aerobic and anaerobic hydrocarbon biodegradation pathways (for reviews see Widdel et al., 2010; Das and Chandran, 2011; Callaghan, 2013a; Heider and Schühle, 2013).

Following the DWH spill, high levels of PAH compounds (>24,000 μg/kg) were detected in deep-sea sediments near the wellhead compared to distant cores (~50 μg/kg), confirming a greater exposure of the resident microflora to aromatic hydrocarbons near the DWH well (OSAT-I, 2010). Metagenomic analysis and targeted functional gene assays of subsurface (1.5–3 cm) deep-sea sediment cores from September to October 2010 revealed increased levels of deltaproteobacteria and genes associated with the anaerobic degradation of aliphatic and aromatic hydrocarbons (e.g., *bssA*, benzoyl-CoA reductase genes, and *assA*) in the sediments located near the well (within 3 km) compared to a distant (128 km) control sample (Kimes et al., 2013). The detection of benzylsuccinate metabolites in the two contaminated sediment cores provided further evidence for the anaerobic biodegradation of alkylbenzenes during this time period. Although there was also genomic evidence for the presence of aerobic hydrocarbon-degrading bacteria, including Oceanospirillales, the abundances were much lower and did not vary between the contaminated and non-contaminated sites (Kimes et al., 2013).

The deep-sea surface sediments (0–1 cm) revealed an increased abundance in gammaproteobacteria, particularly an uncultured gammaproteobacterium and a *Colwellia* sp., in the sediments that demonstrated high levels of hydrocarbon contamination (Mason et al., 2014b). Both 16S rRNA OTUs shared high similarity with previously published sequences from the DWH plume (Valentine et al., 2010; Kessler et al., 2011b; Redmond and Valentine, 2012), from which the *Colwellia* strain was shown to degrade and incorporate a myriad of hydrocarbon compounds (Bælum et al., 2012; Redmond and Valentine, 2012). Nonetheless, Mason et al. (2014b) argue that the surface sediment communities, particularly those with high hydrocarbon concentrations, were distinct in their phylogenetic makeup and functional capacity for hydrocarbon degradation (i.e., dominated by both aliphatic and aromatic hydrocarbon degradation processes) compared to those of the DWH plume. In comparison to the non-contaminated samples, the contaminated surface sediment communities revealed an increased abundance of genes involved in degrading both aliphatic and simple aromatic hydrocarbon compounds (Mason et al., 2014b). Nitrogen cycling in these marine sediments was also impacted by the introduction of aromatic hydrocarbons, which resulted in increased evidence for denitrification (Scott et al., 2014). These deep-sea sediment studies suggest that microbial communities associated with the surface and subsurface environments both have the potential to degrade aliphatic and aromatic hydrocarbons, albeit by distinct dominant processes (i.e., aerobic processes in surface sediments vs. anaerobic processes in subsurface sediments).

Surface sediment samples from two sites, located 2 and 6 km from the DWH wellhead, 1 year after the DWH oil spill revealed a very different bacterial community compared to the surface sediments collected in contaminated sites closer to the DWH well in October/September 2010. The microbial communities in May 2011 displayed diverse bacterial compositions reminiscent of those associated with natural seeps containing relatively high abundances of *Methylococcus*, *Methylobacter*, *Actinobacteria*, *Firmicutes*, and *Chlorofexi* (Liu and Liu, 2013). In addition, the dominant methanotrophs, *Pseudomonas*, *Vibrio*, *Flavobacteria*, and *Acidobacteria* identified by Liu and Liu (2013) were not observed in the Mason et al. (2014b) study. One explanation for this discrepancy could be a site-specific difference in the level of hydrocarbon contamination associated with the two sites. Alternatively, the differences could be indicative of a similar phenomenon in the sediments to that of the DWH plume in which there is a successive change in dominant taxa as a response to the changing hydrocarbon input.

### Coastal sediments/sands

Despite numerous attenuating processes, oil released during the DWH spill was also observed in coastal environments as pooled oil on the surface, as well as tar balls (sometimes referred to as water-in-oil emulsions or mousses) and droplets that settled in coastal sediments and sands (Kostka et al., 2011; Aeppli et al., 2012; Beazley et al., 2012; Boopathy et al., 2012; Liu et al., 2012; Kiruri et al., 2013; Liu and Liu, 2013; Ruddy et al., 2014; Elango et al., 2014; Kappell et al., 2014; Lamendella et al., 2014). The long-term impact of hydrocarbon contamination on coastal ecosystems can vary greatly depending, in part, on the amount of weathering that occurs prior to reaching the coastal environments (Mendelssohn et al., 2012). In the case of the DWH oil spill, chemical analysis of oil mousses sampled from May to July 2010 showed that the oil had already undergone extensive weathering, including the reduction of C9-C16 *n*-alkanes (0.3–1.6% of total composition compared to 54% in MC252 oil) and BTEX/C3-benzenes (an order of magnitude lower levels) in all of the mousses (Liu et al., 2012). PAH concentrations were also altered with a loss of the dominant MC252 PAH, naphthalene, which was reduced to 3-9% of total PAHs compared to 64% in unaltered MC252 oil. A long-term monitoring study (May 2010 to November 2011) of the surface slicks and contaminated beach sands revealed similar findings of highly weathered hydrocarbon compounds associated with the beach sands, and it also detected high-levels (10X higher than in MC252) of oxygenated hydrocarbons, called “**oxyhydrocarbons**,” associated with beach sands (Aeppli et al., 2012). The weathering patterns suggested that photooxidation, evaporation and dissolution were predominantly responsible for the changes in oil composition during the first few months (Aeppli et al., 2012; Liu et al., 2012), while extensive biodegradation occurred during the following year (Aeppli et al., 2012). More extensive and targeted mass spectrometry studies of oxyhydrocarbons associated with oiled sands from Pensacola Beach identified additional oxygen-containing functionalities, including complex ketone, hydroxyl, and carboxylic acid classes of molecules (Ruddy et al., 2014). In addition, tar balls sampled from coastal beaches exhibited the presence of environmentally persistent free radicals, suggesting that aromatic compounds were further transformed through oxidation with transition metals (Kiruri et al., 2013). These data clearly show that the hydrocarbons contaminating coastal ecosystems are highly weathered through multiple processes. However, it should be noted that these processes have also been shown to be variable depending on where the contaminants are deposited (Elango et al., 2014). Although it has long been assumed that weathered hydrocarbons were less toxic due to their decreased bioavailability, the accumulation of weathered byproducts, including oxyhydrocarbons, free radicals, and metals, may present a previously unrecognized toxicity that requires further investigation (Aeppli et al., 2012; Kiruri et al., 2013; McGenity, 2014).

KEY CONCEPT 4 **Oxyhydrocarbons**

Weathering is a physical or chemical process by which hydrocarbon compounds are broken down by natural processes. In one form of weathering, oxygen from the surrounding environment is incorporated into the hydrocarbon compounds, forming oxygenated hydrocarbons, most recently referred to as “oxyhydrocarbons.” Although little is known regarding oxyhydrocarbons, these compounds appear to be recalcitrant and represent the major component of tar balls.

The accumulation of weathered hydrocarbons along the Gulf of Mexico coast caused distinct responses within the coastal sand- and sediment-associated microbial communities. There was a ten-fold increase in the abundance of microbes in contaminated sands, with *Alcanivorax* spp. blooming to 10% of the total community by early July 2010 before falling to <1% in September 2010 (Kostka et al., 2011). Both pyrosequencing and isolation experiments implicated a number of gammaproteobacteria (*Alcanivorax*, *Marinobacter*), and to a lesser extent alphaproteobacteria (*Rhodobacteraceae*) groups, as the driving force behind the increased abundance (Kostka et al., 2011; Lamendella et al., 2014). These findings were consistent with the microorganisms isolated from contaminated soils and metagenomic and metatranscriptomic studies revealing the enrichment of *Rhodobacteraceae* in samples with high levels of hydrocarbon compounds (Lamendella et al., 2014). Furthermore, GeoChip microarray analyses of oiled sands revealed a direct correlation between the relative abundances of hydrocarbon-degrading genes and the level of contamination observed (Beazley et al., 2012; Kappell et al., 2014). Many of the bacteria described in these studies, particularly the isolated strains, are capable of aerobically degrading hydrocarbons, providing a rapid and robust response capable of dealing with the lower weight aliphatic and aromatic compounds present (Kostka et al., 2011; Chakraborty et al., 2012; Overholt et al., 2013). There is also evidence that the microbial communities contain known anaerobic bacteria and their associated functional genes (Beazley et al., 2012; Overholt et al., 2013) and are capable of performing anaerobic hydrocarbon degradation (Boopathy et al., 2012; Overholt et al., 2013).

## Lessons learned—present perspective

The DWH spill has been described as unique (Kujawinski et al., 2011; Peterson et al., 2012) due to the depth at which it occurred, the duration of the incident, the chemical nature of the oil and gases spilt, as well as the large amount of hydrocarbons released (Camilli et al., 2010; Hazen et al., 2010; Atlas and Hazen, 2011; Joye et al., 2011b; Gutierrez et al., 2013). At ~1500 meters in the aphotic marine abyss, the combination of high hydrostatic pressures, low ambient temperatures and the relatively poor nutritional status of the ocean at these depths, led to many questions about the environmental fate of the DWH hydrocarbons, the toxicity of the accidentally and intentionally released organic chemicals and their transformation products, as well as the overall response of the ecosystem to the sudden influx of hydrocarbons and dispersant. Where would the oil go, and what life forms would be impacted? Are microorganisms previously known to metabolize hydrocarbons present within the deep ocean? Can they function under the prevailing environmental conditions? What lessons from other oil spills could be extrapolated to the DWH incident? More specifically, would the metabolic responses of the indigenous microflora be similar to the patterns encountered with the all-too-frequent experience of spills in other environments? Would natural microbial remediation activities be overwhelmed by the conditions in the deep ocean or the toxicity of the oil/dispersant mixtures? Would aerobic heterotrophic microbial respiration of the hydrocarbons lead to the localized depletion of oxygen and the development of anaerobic conditions? Would hydrocarbon decay slow or cease when oxygen is no longer available? Researchers sought to answer some of these major questions following the DWH oil spill, providing us with a wealth of new data and many important insights.

Even a cursory look at the available evidence reveals that not only were the indigenous microflora present and capable of responding to the influx of the hydrocarbons released during the DWH incident, but they also responded quickly and efficiently. This is evident from the integration of isotopic labeling studies and genomic, transcriptomic, and metabolite profiling information (Table S1, Figure 2) that were collected with time and interpreted relative to background areas, which can arguably be considered baselines for comparative purposes. The microbial communities exhibited succession patterns wherein the diversity and complexity normally inherent in the Gulf of Mexico environmental compartments diminished to the point where hydrocarbon-degrading microorganisms were remarkably, but transiently, enriched. Thus, some well-known hydrocarbon-degrading microorganisms, as well as some other not so well known organisms (e.g., *Oceanospirillales, Cycloclasticus, Colwellia*), were able to proliferate at the expense of the large influx of labile hydrocarbons.

One of the more unique features of the DWH incident was the formation of a hydrocarbon and dispersant plume within the water column. The succession of microorganisms in this amorphous habitat has been documented (Kostka et al., 2011; Mason et al., 2012; Redmond and Valentine, 2012; Yang et al., in press), and it is clear that particular types of hydrocarbonoclastic microorganisms were enriched relative to pre-spill conditions. The cold *in situ* temperatures, speculated to restrict natural hydrocarbon biodegradation processes, proved not to be a major factor limiting the fate of the oil (Redmond and Valentine, 2012). Similarly, there was little evidence of a devastating oxygen depletion zone in oil-impacted areas, but only mild and rather consistent oxygen anomalies associated with the biodegradation of hydrocarbon components (Kessler et al., 2011b). In fact, functional gene assays suggested the enrichment of *alkB* (codes for an alkane-1-monooxygenase) and *nahA* (codes for naphthalene-1,2-dioxygenase) genes (as well as many other genes) within the plume (Lu et al., 2012), and these enzymes play well-established and predominant roles in the initial oxidation of *n*-alkane and naphthalene substrates, respectively. However, it should be noted that the naphthalene-1,2-dioxygenase is one of the most diverse enzymes known, and it can catalyze the transformation of up to 76 different substrates via dioxygenation, monooxygenation, desaturation, *O*- and *N*-dealkylation, or sulfoxidation (Resnick et al., 1996). Interestingly, Lu et al. (2012) also noted the enrichment of various genes associated with anaerobic pathways within the oil and dispersant plume, including *bbs* genes that encode proteins involved in beta-oxidation of benzylsuccinate. The latter is formed during anaerobic toluene biodegradation and used as a signature metabolite (Elshahed et al., 2001).

Despite the complex microbial community succession patterns and recovery associated with the DWH incident, the metabolic patterns associated with this oil spill were not drastically different from other oil spills. Characteristic changes occurred to the spilt oil, including the loss of a diverse range of *n*-alkanes as well as low molecular weight aromatic hydrocarbons (Aeppli et al., 2012). These findings are not surprising since metabolism tends to be a unifying feature of life with diverse life forms exhibiting remarkably similar metabolic patterns. In this sense, the DWH oil spill was not unique. This suggests that marine ecosystems, despite their differences, display a similar capacity to respond to the influx of oily hydrocarbons. Thus, with respect to future oil spills, it can reasonably be expected that the resident microbial communities will metabolize the more labile hydrocarbon components, while leaving behind the more recalcitrant materials (e.g., high molecular weight polynuclear aromatic hydrocarbons, resins, and asphaltic components).

Despite the importance of microbial hydrocarbon degradation, it represents only a fraction of the complex oil weathering process (Fingas, 2011; Liu et al., 2012). A recent study noted an increased degree of oxygenation of the DWH oil components, the progressive removal of the saturated and aromatic fractions, and the aggregation of the remaining material with sand to form sand patties that, as of this writing, continue to be deposited on the shores of the Gulf of Mexico (Aeppli et al., 2012). It is clear that the combined impact of weathering processes results in the incorporation of oxygen into the molecular structure of oily components to form the operationally defined “oxyhydrocarbons” (Aeppli et al., 2012). This oxygenated fraction in the sand patties is >50% of the mass of weathered oil and consists of a wide variety of fatty acids and alcohols amongst other constituents. These findings suggest that in assessing future oil spills, researchers should include understanding the microbial metabolism and environmental fate and toxicity of this weathered, oxygenated fraction of oil in their efforts to develop new and effective mitigation approaches.

## Conclusion—future perspective

In a world heavily dependent upon the use of fossil energy (Odell, 2000, 2013), it is inevitable that oil spills will occur. Knowledge gained from the DWH spill in the Gulf of Mexico, however, reinforces that prevalent and well-studied attenuation mechanisms will partially ameliorate the impact of similar environmental catastrophes and that focus on lesser-known hydrocarbon degradation pathways is still needed for future spill assessment and remediation strategies. Studies resulting from the DWH event have demonstrated that it would be wise to shift scientific attention to the environmental impact of the more recalcitrant fraction of oil in order to understand the metabolic fate of the weathered, oxygenated oil components (oxyhydrocarbons) that persist after a spill.

## Note added in review

During the peer-review of this article, two additional reviews were published (Joye et al., 2014; King et al., 2014). Similar to our evaluation of the literature, both articles address the microbial response to the Deepwater Horizon oil spill, but each has unique insights that we highlight here. King et al. (2014) provide a thorough assessment of the mass and physical nature of the hydrocarbons released and their behavior in relation to seawater circulation patterns in the Gulf, as well as the tar balls and tar mats associated with coastal ecosystems. They contrast the microbial community composition in the Gulf both pre- and post-spill. Their review also includes an examination of the fate of dispersants, highlighting their impact on the dynamics of plume bacteria and the degradation of certain hydrocarbons. The Joye et al. (2014) review includes a focus on the formation of marine snow as a mechanism by which surface-derived oil is deposited in both surface and deep-sea sediments. This phenomenon has important implications for the fate of oil and the dynamics of microbial communities mediating hydrocarbon transformations in both the water column and in sediments. Our review includes a focus on the mechanisms involved in the microbial transformation of hydrocarbons and a discussion regarding “lessons learned,” with an emphasis on the importance of “oxyhydrocarbons” in future research. Collectively, the three articles provide an overall consensus of the microbial response to the oil spill and suggest productive avenues for future research.

### Conflict of interest statement

The authors declare that the research was conducted without any commercial or financial relationships that could be construed as a real or perceived conflict of interest.
