# Supplementary material for: Microbial transformation of the Deepwater Horizon oil spill—past, present, and future perspectives
Source: Front Microbiol. 2014 Nov 18;5:603. doi: 10.3389/fmicb.2014.00603 (PMC4235408; doi:10.3389/fmicb.2014.00603)
Supplement: Supplementary file 1 [file Table1.PDF]

Microbial transformation of the Deepwater Horizon oil spill – past, present and future perspectives

Nikole E. Kimes[1,2*], Amy V. Callaghan[3], Joseph M. Suflita[3] and Pamela J. Morris[4]

[1]Evolutionary Genomics Group, División de Microbiología, Universidad Miguel Hernández, San Juan, Alicante, Spain
[2]Current Affiliation: Department of Medicine, University of California, San Francisco, CA, USA
[3]Department of Microbiology and Plant Biology, University of Oklahoma, Norman, OK, USA
[4]Belle W. Baruch Institute for Marine and Coastal Sciences, University of South Carolina, Georgetown, SC, USA

Correspondence:
Dr. Nikole E. Kimes
Department of Medicine - Division of Gastroenterology
University of California, San Francisco
513 Parnassus Ave, Med Sci S-357
San Francisco, CA 94143-0538
nikolekimes@gmail.com

Table S1. Summary of investigations included in this review. (Note – Some studies utilized data from the Operational Science Advisory Team 2010 report (OSAT-I, 2010), which is not cited separately in the table due to the extensive list of studies conducted and reported. Additionally, some studies did not conduct specific sample analyses, but data are available from other studies for those samples and are indicated in the footnotes.)

Ecosystem Type	Sample Source(s)<br>Field Location or Cruise Information<br>(Date)	Hydrocarbon Analysis of Samples	Phylogenetic Surveys and Analyses	Other Analyses and/or Associated Datasets	Reference
**Water column**	U.S. Coast Guard – Authorized flow-assessment effort (May/June 2010)<br><br>National Deep Submergence Facility's AUV Sentry (June 19-28, 2010)	√	-	Dissolved oxygen/water parameter measurements, water velocity measurements	Camilli et al., 2010
	R/V *Pelican* (May 9-16, 2010)	√	-	Dissolved oxygen/water parameter measurements	Diercks et al., 2010
	R/V *Ocean Veritas* and R/V *Brooks McCall* (May 27 - June 2, 2010)	√	16S rRNA Gene clone libraries, phospholipid fatty acid analysis (PFLA), GeoChip 4.0, PhyloChip	Nutrient analyses, acridine orange direct counts, biodegradation assays, synchrotron radiation-based Fourier transform infrared	Hazen et al., 2010

				(SR-FTIR) spectromicroscopy, scanning electron microscopy (SEM)	
	R/V *Cape Hatteras*: PLUMES (Persistent and Localized Underwater Methane Emission Study) (June 11 to 21, 2010)	√	16S rRNA Gene clone libraries	Dissolved oxygen measurements, ($^{13}C/^{12}C$) measurements of methane, ethane and propane via continuous flow isotope ratio mass spectrometry (IRMS), depth contouring, methane oxidation rate measurements, ^{13}C-tracer studies with ethane and propane, hydrocarbon anomaly calculations, hydrocarbon emission estimates	Valentine et al., 2010
	R/V *Walton Smith* (May 25 to June 6, 2010)	√	-	Dissolved oxygen/water parameter measurements	Joye et al., 2011b
	National Oceanic and Atmospheric Administration ship *Pisces* (Aug. 18 to Sept. 2; Sept. 7 – 17; and Sept. 22 to Oct. 4, 2010)	√	16S rRNA gene clone libraries, *pmoA* gene clone libraries	Dissolved oxygen/water parameter measurements, hydrocarbon emission estimates, quantification of methane oxidation rates	Kessler et al., 2011b<br><br>(also see comments on Kessler 2011b by Joye et al., 2011a and Kessler et al., 2011a)
	R/V *Ocean Veritas* (June 6, 2010) (Uncontaminated water)<br><br>Discovery Enterprise drillship (May 22, 2010) (Macondo (MC252) oil)	√[1]	16S rRNA Pyrosequencing	Dissolved oxygen/water parameter measurements[1], enrichment cultures with site material, isolation of microorganisms, hydrocarbon and COREXIT 9500 degradation experiments using *Colwellia* sp. strain RC25, scanning electron microscopy (SEM), synchrotron radiation-based Fourier transform infrared (SR-FTIR) spectromicroscopy of flocs	Bælum et al., 2012

	R/V *Ocean Veritas* and R/V *Brooks McCall*<br>(May 27 - June 2, 2010)	√ [1]	GeoChip 4.0	Dissolved oxygen/water and oil parameter measurements[1]	Lu et al., 2012
	R/V *Ocean Veritas* and R/V *Brooks McCall*<br>(May 27 - June 2, 2010) (Water column)<br><br>Discovery Enterprise drill ship (May 22, 2010)<br>(Macondo (MC252) oil)	√	Metagenomics, metatranscriptomics, single-cell sequencing	Synchrotron radiation-based Fourier transform infrared (SR-FTIR) spectromicroscopy, acridine orange direct counts	Mason et al., 2012
	R/V *Ocean Intervention III* (*Millennium 42* ROV)<br>(June 21, 2010) (Macondo well)<br><br>R/V *Endeavor*: Natural Resource Damage Assessment program (Cruise 478)<br>(June 19-28th, 2010) (Water column)	√	-	-	Reddy et al., 2012
	R/V *Walton Smith*<br>(May 26 - June 5, 2010) (Surface water)<br><br>R/V *Cape Hatteras* (June 11–21, 2010) (Water column)<br><br>National Oceanic and Atmospheric Administration ship *Pisces* (Sept. 7–17, 2010) (Water column)	-	16S rRNA Gene clone libraries, stable isotope probing (SIP)/terminal restriction length polymorphism analysis (TRFLP)	Dissolved oxygen/water parameter measurements, enrichment cultures with water column samples	Redmond and Valentine, 2012
	Pre-spill:<br>R/V *Cape Hatteras* GulfCarbon 5 cruise<br>(March 10 - 21, 2010)<br><br>Post-spill:<br>R/V *Ocean Veritas* (May 25 to June 11, 2010)<br><br>R/V *Brooks McCall* (May 29 to June 27, 2010)<br><br>R/V *Ferrel* (July 3 to August 29, 2010)	√[2]	PhyloChip	Dissolved oxygen/water parameter measurements[2], stable carbon isotope ratios ($^{13}C/^{12}C$) of dissolved methane, enrichments with site material, isolation of microorganisms	Dubinsky et al., 2013
	R/V *Pelican* (May 5, 2010) (Surface water)<br><br>R/V *Walton Smith* (May 31, 2010) (Water column)<br><br>R/V *Pelican* (Sept. 12, 2010) (Water column)<br><br>R/V *Cape Hatteras* (Oct. 18, 2010) (Water column)	-	16S rRNA Pyrosequencing, stable isotope probing (SIP)/clone libraries/qPCR/DGGE	Enrichments with site material, isolation of microorganisms, ^{14}C-mineralization experiments with site water	Gutierrez et al., 2013
	R/V *Ocean Veritas* and R/V *Brooks McCall*<br>(May 29, 2010) (Proximal plume water sample OV01102/03)	√[3]	Single-cell sorting, whole-genome amplification, Illumina HiSeq sequencing	√[1,3]	Mason et al., 2014a
	Pre-spill:	-	16S rRNA Gene clone	Dissolved oxygen/water	Yang et al., 2014

	R/V *Pelican* (March 10, 2010) (Water column)<br><br>Post-spill:<br>R/V *Pelican* (May 5-9, 2010) (Surface water)<br><br>R/V *Walton Smith* (May 31, 2010) (Water column)<br><br>R/V *Pelican* (Sept. 12, 2010) (Water column)<br><br>R/V *Cape Hatteras* (Oct. 18, 2010) (Water column)<br><br>R/V *Endeavor* (July 3, 2011) (Water column)		libraries, 16S rRNA pyrosequencing	parameter measurements	
**Surface water**	U.S. Coast Guard – authorized flow-assessment effort (May/June 2010)<br><br>National Deep Submergence Facility's AUV Sentry (June 19-28th, 2010)	√	-	Dissolved oxygen/water parameter measurements	Camilli et al., 2010
	R/V *Pelican* (May 9-16, 2010)	√	-	Dissolved oxygen/water parameter measurements	Diercks et al., 2010
	R/V *Ocean Veritas* and R/V *Brooks McCall* (May 27 - June 2, 2010)	√	16S rRNA Gene clone libraries, phospholipid fatty acid analysis (PFLA), GeoChip 4.0, PhyloChip	Nutrient analyses, acridine orange direct counts, biodegradation assays, synchrotron radiation-based Fourier transform infrared (SR-FTIR) spectromicroscopy, scanning electron microscopy (SEM)	Hazen et al., 2010
	R/V *Pelican*: Stations OSS (oil spill site) and CT (control) (May 2010)<br>(Surface water and sea surface oil mousses)<br><br>Marsh Point (MP), Davis Bayou, MS (July 21, 2010)<br>(Oil mousses associated with salt marsh)<br><br>Stations 'Grab' and 'Core' (May 2011) (Sediment)	√	-	Trace metal analysis	Liu et al., 2012
	R/V *Walton Smith*<br>(May 26 - June 5, 2010) (Surface water)<br><br>R/V *Cape Hatteras* (June 11–21, 2010) (Water column)<br><br>National Oceanic and Atmospheric Administration ship *Pisces* (Sep. 7–17, 2010) (Water column)	-	16S rRNA Gene clone libraries, stable isotope probing (SIP)/terminal restriction length polymorphism analysis (TRFLP)	Dissolved oxygen/water parameter measurements, enrichment cultures with water column samples	Redmond and Valentine, 2012

	R/V *Pelican* (May 5, 2010) (Surface water)<br><br>R/V *Walton Smith* (May 31, 2010) (Water column)<br><br>R/V *Pelican* (Sept. 12, 2010) (Water column)<br><br>R/V *Cape Hatteras* (Oct. 18, 2010) (Water column)	-	16S rRNA Pyrosequencing, stable isotope probing (SIP)/clone libraries/qPCR/DGGE	Enrichments with site material, isolation of microorganisms, ^{14}C-mineralization experiments with site water	Gutierrez et al., 2013
	R/V *Pelican*: Stations OSS (oil spill site) and CT (control) (May 2010)<br>(Surface water without visible oil and sea surface oil mousses)<br><br>Marsh Point (MP), Davis Bayou, MS (July 21, 2010)<br>(Oil mousses associated with salt marsh)<br><br>Stations 'Grab' and 'Core' (May 2011) (Sediment)	√[4]	16S rRNA Pyrosequencing	-	Liu and Liu, 2013
	Pre-spill:<br>R/V *Pelican* (March 10, 2010) (Water column)<br><br>Post-spill:<br>R/V *Pelican* (May 5-9, 2010) (Surface water)<br><br>R/V *Walton Smith* (May 31, 2010) (Water column)<br><br>R/V *Pelican* (Sept. 12, 2010) (Water column)<br><br>R/V *Cape Hatteras* (Oct. 18, 2010) (Water column)<br><br>R/V *Endeavor* (July 3, 2011) (Water column)	-	16S rRNA Gene clone libraries, 16S rRNA pyrosequencing	Dissolved oxygen/water parameter measurements	Yang et al., 2014
**Deep-sea sediments**	R/V *Pelican*: Stations OSS (oil spill site) and CT (control) (May 2010)<br>(Surface water and sea surface oil mousses)<br><br>Marsh Point (MP), Davis Bayou, MS (July 21, 2010)<br>(Oil mousses associated with salt marsh)<br><br>Stations 'Grab' and 'Core' (May 2011) (Sediment)	√	-	Trace metal analysis	Liu et al., 2012
	OSAT-I R/V *Gyre* cruises<br>(Sept. 16 to Oct. 20, 2010)<br>(Contaminated and uncontaminated GOM sediments)	√[5]	Metagenomics, targeted functional gene surveys	Metabolite profiling	Kimes et al., 2013
	R/V *Pelican*: Stations OSS (oil spill site) and CT (control) (May 2010)<br>(Surface water without visible oil and sea surface oil	√[4]	16S rRNA Pyrosequencing	-	Liu and Liu, 2013

	mousses)<br><br>Marsh Point (MP), Davis Bayou, MS (July 21, 2010) (Oil mousses associated with salt marsh)<br><br>Stations 'Grab' and 'Core' (May 2011) (Sediment)				
	OSAT-I R/V *Gyre* cruises<br>(Sept. 16 to Oct. 20, 2010)<br>(Contaminated and uncontaminated GOM sediments)	√	Metagenomics	Nutrient analyses, ^{14}C-mineralization experiments with site sediments, predictive relative metabolic turnover (PRMT) analysis	Mason et al., 2014b
	OSAT-I R/V *Gyre* cruises<br>(Contaminated and uncontaminated GOM sediments)<br>(Sept. 16 to Oct. 20, 2010)<br><br>Coal Oil Point, Santa Barbara Channel, CA<br>(2008 – 2010) (Natural oil seep samples)	√[6,7]	Comparative metagenomic analysis[6,8]	Sediment nutrient analysis[6]	Scott et al., 2014
**Coastal sediments, beach sands, salt marshes, and tar balls**	Pre-spill:<br>St. George Island, FL<br>(April 2010) (Beach sands)<br><br>Post-spill:<br>Discovery Enterprise drillship<br>(May 22, 2010) (Macondo (MC252) oil)<br><br>Pensacola Beach, FL<br>(July 2, July 30, and Sept.1, 2010) (Beach sands)	√	Automated ribosomal intergenic spacer analysis (ARISA), PCR of near full length SSU rRNA, DNA- and RNA-based pyrosequencing of SSU rRNA, qPCR of SSU rRNA and *Alcanivorax*	Enumeration (MPN), enrichment, and isolation of oil-degrading bacteria, phenotypic MicroArray (PM) analysis of isolates	Kostka et al., 2011
	Macondo (MC252) well oil<br><br>Surface slicks (including oil scraped off freshly oiled marsh grasses and Deepwater Horizon debris)<br>(May 31, 2010 – June 11, 2010)<br><br>Perdido Beach, FL, Gulf Shores, AL Fort Morgan, AL, Dauphin Island, AL, Gulf Port, MS, Waveland, MS Chandeleur Islands, LA, Grand Isle, LA:<br>(July 2, 2010 – Nov. 28, 2011) (Sand patties)<br>(April 6, 2011 – July 19, 2011) (Rock scrapings)	√	-	Elemental analysis	Aeppli et al., 2012
	Eastern side of the Point Aux Pins peninsula, AL<br>(June 8, July 2, and Sept. 10, 2010)<br>(Sediment cores)<br><br>Eastern side of the Point Aux Pins peninsula, AL	√	G3 PhyloChip, GeoChip 2.0	Dissolved oxygen and site-water parameter measurements	Beazley et al., 2012

	(July 2, 2010)<br>(Tar balls and tar mats mixed with wrack (organic debris))				
	Barataria Bay, LA<br>(Marsh sediments)	√	-	Sediment characterization, biodegradation experiments, bacterial plate counts of cultures	Boopathy et al., 2012
	R/V *Pelican*: Stations OSS (oil spill site) and CT (control)<br>(May 2010)<br>(Surface water and sea surface oil mousses)<br><br>Marsh Point (MP), Davis Bayou, MS (July 21, 2010)<br>(Oil mousses associated with salt marsh)<br><br>Stations ‘Grab’ and ‘Core’ (May 2011) (Sediment)	√	-	Trace metal analysis	Liu et al., 2012
	Gulf Shores, AL<br>(May1, June 15, and July 21, 2010) (Tar balls)<br><br>Pensacola Beach, FL<br>(July 14, 2011) (Tar balls)	√	-	Radical decay kinetic experiments, EPR to determine presence of environmentally persistent free radicals (EPFRs), OH radical generation studies	Kiruri et al., 2013
	R/V *Pelican*: Stations OSS (oil spill site) and CT (control)<br>(May 2010)<br>(Surface water without visible oil and sea surface oil mousses)<br><br>Marsh Point (MP), Davis Bayou, MS (July 21, 2010)<br>(Oil mousses associated with salt marsh)<br><br>Stations ‘Grab’ and ‘Core’ (May 2011) (Sediment)	√[4]	16S rRNA Pyrosequencing	-	Liu and Liu., 2013
	Elmer’s Island Beach, LA<br>(June 3, 2010) (Beach sediment)<br><br>Pensacola Beach, FL<br>(July 30, 2010) (Beach sediment)	√[9]	Illumina HiSeq sequencing of isolates	Nutrient analyses and phenotypic characterization of isolates[9]	Overholt et al., 2013
	Caminada Headlands Beach , LA (5 sites)<br>Fourchon Beach, LA (4 sites)<br>Elmer’s Island Beach, LA (1 site)<br><br>(October 26, 2010 – May 31, 2012)<br>(Submerged oil mats (SOM), snare oil, and surface residue balls (SRBs))	√[10]	-	Nutrient and moisture content analysis (this study); physical, chemical and microbiological parameters[10]	Elango et al. 2014
	Cotton Bayou Beach Area, Orange Beach, AL<br>(June 13 – 15, 2010, August 9, 2010, Sept. 20- 22, 2010;	√[11]	GeoChip 4.2, Illumina sequencing of bacterial	Enrichments with site material	Kappell et al., 2014

	Nov. 15 -18, 2010, and August 15 – 17, 2011) (Surface sand)<br><br>St. George Island, FL<br>(June 13 – 15, 2010, Sept. 20- 22, 2010; Nov. 15 -18, 2010, and August 15 – 17, 2011)) (Surface sand)		16S rRNA genes		
	Elmer's Beach, LA<br>(June 3, 2010, June 21, 2010, and June 29, 2010)<br>(Beach sand cores)	√	Pyrosequencing of SSU rRNA, metatranscriptomics	Acridine orange direct counts, enrichments with site material, isolation of microorganisms, oil and COREXIT 9500 biodegradation assays using *Marinobacter* isolate33	Lamendella et al., 2014
	Santa Rosa Island near Pensacola Beach, FL<br>(June 30, 2010) (Sand)<br><br>Saint George Island, FL<br>(June 30, 2010) (Sand)	√	-	-	Ruddy et al., 2014

[1]Metadata obtained from Hazen et al., 2010
[2]Some hydrocarbon and oxygen data were compiled from the NOAA-National Oceanic Data Center archive of data observations made aboard research survey vessels supporting the Subsurface Monitoring Unit in the Gulf of Mexico (www.nodc.noaa.gov/General/DeepwaterHorizon/ships.html)
[3] Metadata from Mason et al., 2012
[4] Metadata obtained from Liu et al., 2012
[5] Metadata obtained from OSAT-I, 2010
[6] Metagenomic and metadata obtained from Mason et al., 2014b
[7] Metadata obtained from Lorenson et al., 2011
[8] Metagenomic data obtained from Hawley et al. 2014
[9]For sampling information and metadata, see Kostka et al., 2011
[10]Includes additional data from and comparison to data obtained from Urbano et al., 2013, Lemelle et al. 2014, and/or OSAT-II 2011.
[11]Metadata from Newton et al., 2013
